# Tips and Tricks in surgical reduction of the posterior column of AO/OTA C3 pilon fractures

**DOI:** 10.1186/s12891-021-04890-6

**Published:** 2022-01-03

**Authors:** Moran Huang, Qiuke Wang, Junjie Guan, Kexin Liu, Yunfeng Chen, Lei Wang

**Affiliations:** Department of Orthopedic Surgery, and Shanghai Institute of Microsurgery on Extremities, Shanghai Jiao Tong University Affiliated Sixth People’s Hospital, Shanghai, China

**Keywords:** Pilon fractures, Posterior column reduction, Surgical approach, Treatment outcome, Die-punch fragment

## Abstract

**Background:**

Accurate posterior column reduction remains a challenging and controversial topic in the management of complex pilon fractures (AO/OTA C3). We aim to report the outcomes of surgical treatment for 22 AO/OTA C3 pilon fracture cases between January 2015 and May 2017 and highlight some traps and tips.

**Methods:**

Three patients underwent two-stage early plating on the posterior column through a posterolateral approach. The remaining 19 patients were treated with two-stage delayed plating on the posterior column: 11 patients were treated with a posterolateral approach, five patients with a modified posteromedial approach, and three patients with a single anterior approach. The reduction of the posterior column was evaluated according to the Burwell-Charnley’s radiographic criteria, and functional outcomes were assessed using the American Orthopedic Foot and Ankle Society (AOFAS) scores.

**Results:**

Posterior column malreduction occurred in five cases, including in one case that was re-adjusted immediately and in another case that was re-adjusted during a two-staged delayed operation. According to Burwell-Charnley’s criteria, the satisfactory rate of fracture reduction was 81.8%. After 1 year, the mean AOFAS score was 81.9 (81.9 ± 9.9); the outcome was excellent in three (20.0%), good in nine (60.0%), and fair in three (20.0%). Excellent or good outcomes were noted in 12 patients (80.0%).

**Conclusions:**

The combined anterior and posterior approach is suggested in the second stage of plating so that the posterior column fragments can be re-adjusted intraoperatively, if necessary. Following these procedures, satisfactory reduction and recovery of good ankle function can be anticipated.

## Background

Pilon fractures account for 4 to 10% of tibial and ankle fractures [1] and often present a challenge to the foot and ankle surgeon. According to AO Foundation/Orthopaedic Trauma Association (AO/OTA) classification [2], C3 type pilon fracture is defined as a comminuted fracture of the articular surface of the distal tibia, involving the metaphysis and diaphysis, and is often accompanied by severe soft tissue injury. Surgical treatment is commonly indicated for these fractures. Compared to other techniques (i.e. intramedullary nail), the locking plate has advantages in terms of fracture reduction [3]. Owing to the high failure rate of early tibial open reduction and internal fixation (ORIF), Sirkin [4] and Patterson [5] reported a two-stage surgical protocol, in which tibial external fixation combined with fibular internal plate fixation is performed in the first stage. Definitive internal fixation is completed in the second stage after the reduction of soft tissue swelling, and open wound infection is controlled. Since a lower rate of postoperative complications using the two-staged protocol was reported, the concept has gradually been promoted and applied in real practice.

Since the posterior column articular surface can serve as a reference for the final reduction on the articular surface of distal tibial plafond, posterior column reduction is widely preferred [6]. For the treatment of type C2 and C3 pilon fractures, Ketz et al. [7] adopted a staged treatment including posterior column plating at the first stage, with satisfactory clinical outcomes. However, 5 years later, Sanders et al. [8] analyzed more cases of type C pilon fractures and found that the quality of reduction was not significantly improved by posterior column plating at an early stage. The different conclusions from the two studies could be explained by the different proportions of each AO/OTA classification type between the two individual samples. Overall, the reduction strategy of the posterior column remains controversial, especially for complicated C3 pilon fractures.

In this study, we reviewed the surgical outcomes of complex (AO/OTA C3) pilon fractures treated using a two-staged protocol in a single trauma center. We aimed to report the results and clinical outcomes of posterior column reduction, highlight common pitfalls, and offer novel insights.

## Methods

### Study design

We reviewed 22 AO/OTA C3 pilon fractures with ORIF observed at our level I trauma center between January 2015 and May 2017. The inclusion criteria were: (1) age of 18 years or older; (2) minimum 12-month follow-up; (3) complete clinical, functional, and imaging data; (4) treatment by ORIF; and (5) normal ankle function before injury.

Radiography and computed tomography (CT) of the ankle joint were routinely performed before surgery. All patients were treated with a two-stage protocol, including debridement within 10 h for all open fractures at the emergency department. The first-stage treatment consisted of splint, calcaneal traction, and external fixation with or without posterior column plating. The second-stage intervention consisted of definitive ORIF after resolution of the soft-tissue injury. Early plating indicated that the posterior column was plated in the first stage, while late plating suggested that the posterior column was plated in the second stage. All methods were performed in accordance with relevant guidelines and regulations.

### Surgical technique

Under general anesthesia, patients underwent a single anterior approach or combined modified posteromedial and anterolateral approaches in the supine position. Those undergoing a combined posterolateral and anterior extensile approach were placed in the floating position. A tourniquet was placed on the thigh, and the extremity was prepped and draped in a sterile fashion.

In the single anterior approach, the articular surface of the distal tibia was exposed anteriorly as far as possible, since the posterior column was not displaced significantly in such cases. The reduction was performed sequentially from posterior to anterior, followed by provisional fixation using Kirschner wires. For the cases with combined approaches, the ORIF of the posterior column was first performed according to the posterior cortex alignment through the posterior approach, and could be adjusted by the combined approaches if necessary. If there was a central impacted fragment in the distal tibial plafond, it should have be released thoroughly through the combined approaches, and the posterior column could have be temporarily instrumented using a plate and without long screws before the anterior plafond was reduced. Subsequent reduction of the entire tibial platfond was accomplished through the anterior approach. In some cases, posterior column malreduction resulted in ankle joint incongruency after the anterior reduction was completed. If the posterior column plating was performed early in the two-stage operation, then the posterior articular fragment would have to be re-adjusted later through the anterior approach, which can be technically challenging. In contrast, when posterior column plating is performed late in the two-stage operation, re-adjustment can be performed immediately through the posterior approach.

As soon as the reduction of every articular fracture was confirmed as satisfactory both by direct viewing and fluoroscopy, an allograft or bone graft substitute was placed. After the correct alignment was confirmed through C-arm radiography, definitive fixation with distal tibial locking plates was performed through the anterior approach on the medial and anterolateral sides. Finally, the anterior and posterior incisions were irrigated and closed.

### Rehabilitation and follow-up

Postoperatively, the ankle joint was immobilized in a neutral position with a posterior splint for 2 weeks, which was beneficial for promoting soft-tissue healing and preventing equinus. Stitches were removed after 3 weeks, and if the incision had properly healed, ankle exercise with a free range of motion was permitted. Depending on the radiological findings, partial weight-bearing was gradually allowed in 4–6 weeks and full weight-bearing was permitted at 10–12 weeks postoperatively.

According to the criteria of Burwell-Charnley [9], the reduction quality was evaluated by CT scans and radiographs taken on the first day after the operation, and the specific criteria was shown in Table 1. During the follow-up, radiography was performed at 4 weeks and at 2, 3, 6 and 12 months postoperatively. During follow-up visits, the patient was carefully examined for signs of complications such as necrosis, infection, delayed union, nonunion, malunion, and failure of fixation. The American Orthopedic Foot and Ankle Society (AOFAS) score [10, 11] was used to assess ankle function at the final visit. The outcomes measurements were assessed by an observer who was blind to treatment protocol. All data were collected and analyzed using SPSS version 21 (IBM Corp, Armonk, NY, USA).

**Table 1:** Displaced ankle fracture radiographic reduction criteria of Burwell-Charnley

Anatomical	No medial or lateral displacement of the medial and lateral malleoli No angulation Not more than 1 mm longitudinal displacement of the medial and lateral malleoli Not more than 2 mm proximal displacement of a large posterior fragment No displacement of the talus
Fair	No medial or lateral displacement of the medial and lateral malleoli No angulation 2–5 mm posterior displacement of the lateral malleolus 2–5 mm proximal displacement of a large posterior fragment No displacement of the talus
Poor	Any medial or lateral displacement of the medial and lateral malleoli More than 5 mm posterior displacement of the lateral malleolus or more than 5 mm displacement of the posterior malleolus Any residual displacement of the talus

## Results

Five patients were lost to follow-up in our study, and we failed to contact them. The sample consisted of 16 (72.7%) men and 6 (27.3%) women with a mean age of 48.5 ± 12.8 (range, 20 to 64) years. Ten patients were injured due to falling from a height and 12 due to vehicle accidents. Six patients had open fractures, two of which were classified as Gustilo grade I and four as grade II. The remaining 16 patients had closed fractures, four of which were classified as Tscherne grade I, 10 as grade II, and 2 as grade III. Six patients had associated injures, including 16 fibular fractures, three craniocerebral trauma, one rib fracture, one ipsilateral talus fractures and ipsilateral distal radius fracture.

All fractures were treated 6 to 40 days after injury, with a mean of 15.9 days. Regarding the surgical procedure, the posterior column was fixed using a two-stage approach, with early plating through the posterolateral approach in three cases, and the mean total operation time was 163.3 (range, 150 to 180) min. For the remaining 19 cases, two-stage delayed fixation was adopted, and the mean total operation time was 107.9 (range, 80 to 190) min. Of those 19 cases, the posterior column was definitively plated through the posterolateral approach in 11 (57.9%) cases, modified posteromedial approach in five cases (26.3%), and a single anterior approach in three (15.8%) cases.

**Fig. 1:**
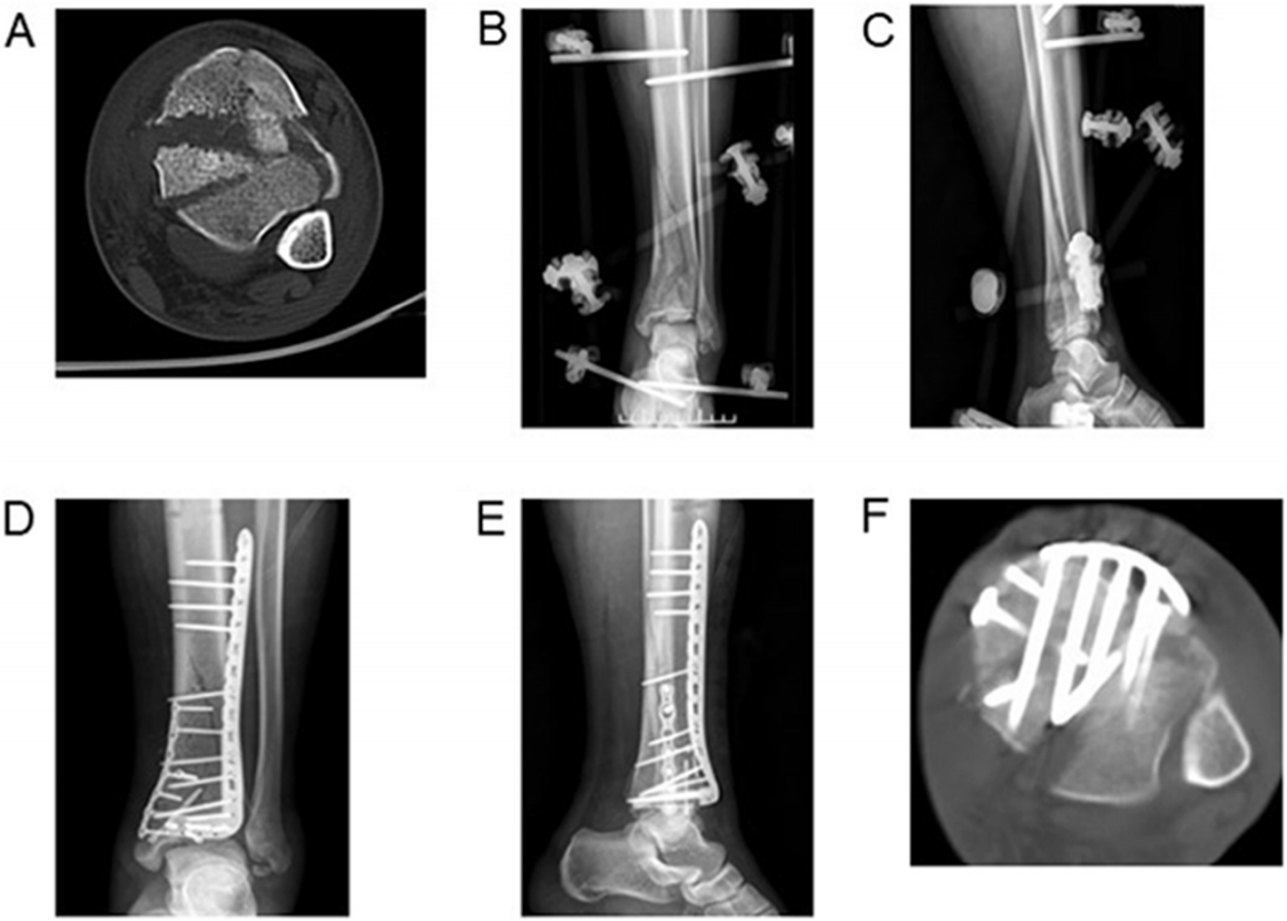
A 20-year-old man involved in a fall developed AO/OTA C3 pilon fracture. **A**) Preoperative transverse computed tomography (CT) scans showed severe fracture displacement of the distal tibial articular surface. **B** & **C**) The anteroposterior and lateral view of the ankle showed that external fixation was used to maintain the length and axial alignment of the limb after undergoing external fixation in the first stage for 19 days. **D** & **E**) The lateral view showed good reduction and fixation after undergoing open reduction and internal fixation through the simple anterior approach in the delayed procedure. **F**) Postoperative transverse CT scans showed articular surface step-off of more than 5 mm

**Fig. 2:**
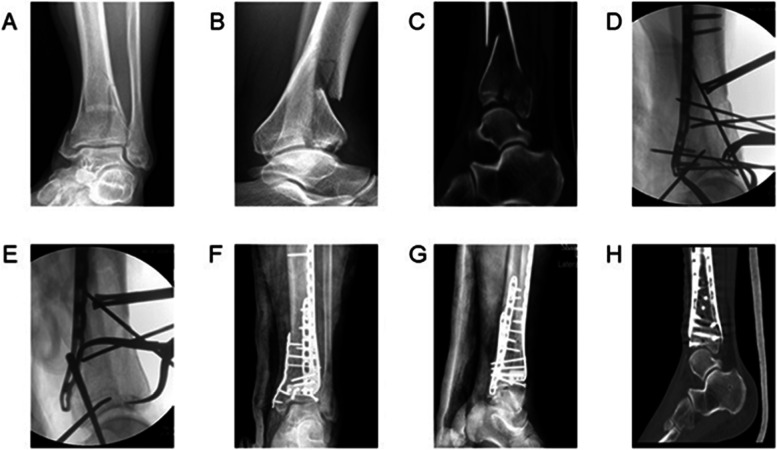
A 52-year-old man was involved in a motor vehicle accident developed AO/OTA C3 pilon fracture. **A**, **B** & **C**) Preoperative anteroposterior and lateral radiographs and sagittal computed tomography (CT) scans showed comminuted fracture of the posterior column articular surface. **D** & **E**) The intraoperative lateral radiographs showed adjustment of the articular surface through the combined posterolateral and anterior extensile approach, before (D) and after (E) the procedure. **F** & **G**) The postoperative anteroposterior and lateral X-rays showed good reduction and fixation. **H**) Postoperative sagittal CT scans showed that the reduction of the articular surface was satisfactory

**Fig. 3:**
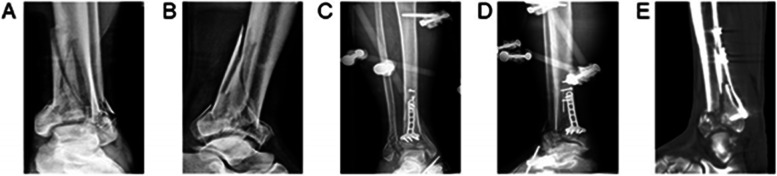
A 52-year-old man involved in a motor vehicle accident developed AO/OTA C3 pilon fracture. **A** & **B**) Preoperative anteroposterior and lateral radiographs showed the fibular fracture and distal tibial articular surface multi-fragment. **C** & **D**) The anteroposterior and lateral view of the ankle showed external fixation was used to maintain the length and axial alignment of the limb undergoing external and internal fixation in the first stage. **E**) The postoperative sagittal computed tomography scans showed that the congruency of the articular surface was not satisfactory after undergoing external and internal fixation in the first stage

**Fig. 4:**
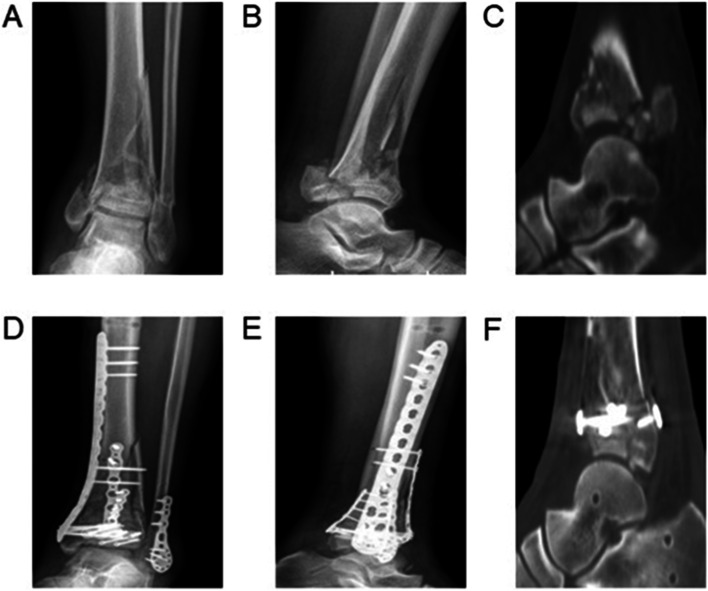
A 58-year-old man involved in a motor vehicle accident developed AO/OTA C3 pilon fracture. **A** & **B**) Preoperative anteroposterior and lateral radiographs showed the fibular fracture and distal tibial articular surface comminuted fracture. **C**) Preoperative sagittal computed tomography (CT) scans showed comminuted fracture of the posterior column articular surface. **D** & **E**) The anteroposterior and lateral radiographs showed good reduction and fixation after open reduction and internal fixation through the combined posterolateral and anterior extensile approach. **F**) Postoperative sagittal CT scans showed that the congruency of the articular surface was not satisfactory

According to the criteria of Burwell-Charnley, the reduction was satisfactory in 18 (81.8%) cases, fair in three (13.6%) cases, and poor in one (4.5%) case. Posterior column malreduction was observed in five cases. In one case, with a single anterior approach during the two-stage delayed operation, the posterior gap of the articular surface was not found on fluoroscopy intraoperatively, but confirmed postoperatively as > 5 mm on a CT scan **(**Fig. 1**)**. In another case with a combined modified posteromedial and anterolateral approaches in a two-stage delayed operation, the articular step-off was more than 2 mm but less than 5 mm postoperatively. For the other three cases with articular surface incongruencies, one case was successfully re-adjusted during the two-stage delayed plating on the posterior column with the combined posterolateral and anterior approaches **(**Fig. 2**)**. The second case could not be anatomically re-adjusted through the anterior approach in the two-staged delayed operation **(**Fig. 3**)**. For the third case, the malreduction went unnoticed owing to hardware overlap in the lateral view during intraoperative fluoroscopy **(**Fig. 4**)**.

Seventeen patients were followed for a mean of 21.9 ± 6.3 (range, 12 to 31) months after surgery, while five patients were lost to follow-up. Nonunion occurred in one case, which eventually achieved union after partial implant removal and autogenous iliac bone graft. One patient developed a deep infection, which was successfully treated with implant removal and debridement. The mean fracture union time of the remaining 15 patients was 3.7 (range, 3 to 6) months. Two patients had a superficial infection, which healed after treatment with oral antibiotics and wound care. Two patients experienced superficial necrosis of the incision corner, which healed after scars fell off approximately 3 months later. The overall mean AOFAS score at the last individual follow-up was 81.9 (81.9 ± 9.9); excellent in three (20.0%) cases, good in nine (60.0%) cases, and fair in three (20.0%) cases; therefore, the rate of excellent and good was 80.0% (12/15) in total. The treatment characteristics are listed in Table 2.

**Table 2:** A statistical description of the case series (*N* = 22 ft in 22 patients)

Approach	Fracture type	Mal-reduction	articular step-off	AOFAS score	Complications	fracture union(m)	follow up(m)
A (*n* = 3)	C3.1	N	<2 mm	/	/	/	/
	C3.1	N	<2 mm	89	/	3	24
	C3.3	Y	>5 mm	83	/	3	12
B (*n* = 5)	C3.1	N	<2 mm	61	/	6	13
	C3.1	N	2-5 mm	83	/	3.5	15
	C3.2	N	<2 mm	/	/	/	/
	C3.3	N	<2 mm	89	/	3	18
	C3.3	N	<2 mm	/	/	/	/
C (*n* = 14)	C3.1	N	<2 mm	89	/	3	24
	C3.1	N	<2 mm	92	Superficial necrosis	3.5	30
	C3.2	Y	<2 mm	90	/	3.5	24
	C3.2	Y	2-5 mm	63	/	3.5	13
	C3.2	N	<2 mm	91	/	4	30
	C3.2	N	<2 mm	66	/	3	18
	C3.2	N	<2 mm	/	/	/	/
	C3.2	N	<2 mm	83	/	4	26
	C3.2	N	<2 mm	87	/	3	24
	C3.3	Y	<2 mm	81	superficial infection	4.5	19
	C3.3	N	<2 mm	/	/	/	/
	C3.3	N	<2 mm	66	nonunion; Superficial necrosis	4	31
	C3.3	N	<2 mm	89	deep infection	4	28
	C3.3	N	<2 mm	81	superficial infection	3.5	23

*Note*: A, B and C represent single anterior approach, modified posteromedial approach and posterolateral approach, respectively

## Discussion

Many factors can influence the outcome of pilon fracture, including fracture classification, reduction quality, soft tissue damage, and whether it was a high- or low-energy injury [12]. For complex pilon fracture, the clinical application of staged treatment has significantly decreased soft tissue complications, resulting in better outcomes [13]. Meanwhile, some scholars hold the opinion that the quality of reduction is the only factor that surgeons could change currently [14], and it is also an essential factor for post-operative rehabilitation (based on results of gait analysis) [15]. However, it is still uncertain how the reduction of the posterior column can be integrated into the staged treatment of complex pilon fracture. Fibula fixation plays an important role in determining the length of the posterolateral key fragment. However, fibula fixation alone cannot manage the anteroposterior angulation or rotation of the posterolateral key fragment. Therefore, the advantages and disadvantages as well as some traps and tips regarding posterior column reduction should be focused on, especially during the staged treatment on complex type C3 pilon fracture.

The posterior column reduction of the pilon fracture depends mostly on the access and view of the anatomical cortical reference of the posterior column. For complex type C3 pilon fractures, posterior column reduction can be challenging in the presence of significant comminuted fractures. Ketz et al. [7] proposed that when malreductions are found after the posterior column is plated in a two stage early operation, they need to be revised immediately or re-adjusted at the later operation. Assal et al. [16] published their preliminary experience with combined modified posteromedial and extensile anterior approach in six cases of type C3 tibial pilon fractures. The posterior column was plated early in the two-staged delayed operation, which resulted in an articular surface step-off of more than 2 mm in four cases, as measured by radiography postoperatively. These results question the reliability of the “anatomic reduction” of the posterior column for type C3 pilon fracture by direct visualization, as some aspects may be neglected during direct reduction through the posterior approach.

According to the Burwell-Charnley reduction criteria, the overall satisfactory rates of definitive reduction on the articular surface of type C3 pilon fractures were 81.8%, as confirmed by CT postoperatively. At the last follow-up, the mean AOFAS score was 81.9 (81.9 ± 9.9), which was ranked excellent in three (20.0%) cases, good in nine (60.0%) cases, and fair in three (20.0%) cases; these results for type C pilon fractures are similar to those reported by Ketz et al. [7].

In the early period of our study, the posterior column tended to be plated in a two-stage early operation. In one of these cases, the articular surface was found to be malreduced postoperatively, which posed a challenge during revision through the anterior approach in the two-stage delayed operation. We believe that in this patient, early posterior column plating was the reason for the malreduction, given the lack of anatomical references due to a comminuted cortex and articular fragments displaced anteriorly. Therefore, the technique was modified in the latter period of the study, using open reduction and plating of the posterior column in the two-stage delayed operation. Such change allowed adjustments through the combined anterior and posterior approaches during the surgery.

A single anterior approach was used in three cases, one of which resulted in an articular surface gap of more than 5 mm after the definitive operation. We proposed that the bone callus around the posterior column fragments could not be thoroughly released through the single anterior approach, which prevented the subsequent reduction procedure on a two-stage delayed operation. In addition, it was difficult to expose the posterior articular surface from anterior to posterior in direct view and to identify the articular gap by C-arm radiography on the lateral view intraoperatively. Therefore, for delayed surgery of type C3 pilon fracture, we do not recommend reducing the posterior column through the anterior approach alone. We suggest that the use of combined anterior and posterior approach is a better option for open reduction of the posterior column in direct view [17]. We had 19 cases in which the combined approach was used; for 16 (84.2%) of them, the postoperative articular surface step-off was less than 2 mm.

There was another three cases with posterior column fragments malreductions, including articular surface incongruency in two cases and step-off in one case. The causes of incongruency included the shadow of prior plating on the lateral malleolus, insufficient angle of intraoperative fluoroscopic image and lack of complete releasing die-punch fragments. For type C3 pilon fractures, articular surface impaction in the central part of the distal tibial plafond, also known as the die-punch fragment, was invariably present. This fragment may cause problems during posterior column reduction if such a procedure is performed conventionally as a first step through the posterior approach. Since the central impacted fragment was not released beforehand, even when the reduction seemed satisfactory based on the limited direct view of the cortical reference posteriorly, the posterior column was pushed backward before posterior plating. In one of the cases, the die-punch fragment was detached and in free style, without offering any resistance when the posterior column was pushed forward by the posterior plating. Traditionally, the posterior column is reduced first as the “keystone” of the whole articular surface reduction of pilon fractures [18]. However, there were still such traps related to articular surface incongruency when die-punch fragments were dealt with inadequately, as observed in the current study. Therefore, we recommend that the posterior column should be fixed with a 2.7-mm system plate, which can be re-adjusted easily through the subsequent anterior approach if necessary.

According to the intraoperative fluoroscopic image in the lateral view of these three cases, the evaluation of the distal tibial articular surface was disturbed by the shadow of prior plating on the lateral malleolus. In the following cases, we chose to perform temporary fixation on the lateral malleolus using Kirshner wires and delay definitive plating until the articular surface reduction was satisfactory, with minimal interference from the implant by fluoroscopy. This technique is recommended for lateral malleolar fractures that are not heavily comminuted.

This study has some limitations. First, there exists an inherent selection bias owing to the retrospective study design. Second, the sample size was relatively small, and the follow-up time was relatively short. Third, there may be a deviation in observer measuring results. Fourth, selection bias cannot be ruled out since we ignored the missing values caused by loss to follow-up. Thus, the results in this study were only valid with the assumption of missing completely at random, which means the lost cases were not related to the observed and unobserved outcomes. However, as this study mainly focused on tips for surgical techniques, the above issue regarding follow-up had limited influence on conclusions. Despite recognizing the limitations of our survey, we believe that the results of this study may be useful in the future development of prospective cohort studies and randomized controlled trials that focus on posterior column reduction of complex pilon fractures. Future research is ongoing and will expand the number of cases and increase the follow-up, providing more statistical strength to the data and better guidance for clinical treatments.

## Conclusions

In summary, this study demonstrated our experience of potential pitfalls and offered recommendations on the posterior column reduction of AOOTA C3 pilon fractures. We suggest a combined anterior and posterior approach in the second stage in order for the posterior column to be reduced and adjusted simultaneously, whenever needed. Following with these procedures, a satisfactory reduction and good recovery of ankle function should be expected.

## Data Availability

The datasets used and/or analyzed during this study are available from the corresponding author on reasonable request.

## Abbreviations

AOFAS: American Orthopedic Foot and Ankle Society scores
CT: Computed tomography
ORIF: Open reduction and internal fixation
